# Non-intubated esophageal underwater endoscopic submucosal dissection: “one-way flow” via stiffened orogastric drainage

**DOI:** 10.1055/a-2854-7000

**Published:** 2026-07-23

**Authors:** Kazuma Fukuzawa, Kazuhiro Furukawa, Takashi Hirose, Hiroyuki Shibata, Yoshiyuki Kurata, Yui Oshitani, Hiroki Kawashima

**Affiliations:** 1 Department of Gastroenterology and Hepatology 36589 Nagoya University Graduate School of MedicineNagoyaJapan; 2 Department of Endoscopy 36590 Nagoya University HospitalNagoyaJapan

Underwater endoscopic submucosal dissection (UESD) enhances submucosal visualization and facilitates precise dissection 1 2 . However, esophageal application is constrained by the risk of fluid regurgitation and aspiration; accordingly, airway protection with endotracheal intubation is often recommended or chosen for upper esophageal/pharyngeal procedures 3 . We present a non-intubated approach that creates a gravity-plus-decompression “one-way flow” using guidewire-stiffened orogastric (OG) continuous drainage.

The patient was a woman in her 80s with a circumferential 5.5-cm squamous cell carcinoma of the upper thoracic esophagus. UESD was performed under conscious sedation using a GIF-H290T endoscope and normal saline. An OG tube was inserted orally (not nasally) at the start of the procedure. We deliberately chose the shorter, straighter oral route and the smaller intraoral space to reduce the redundant tube length and path curvature ( Fig. 1 ); a nasal route creates a longer S-shaped course that is prone to bending, kinking, coiling, and tip displacement when the scope is advanced or withdrawn. To lessen scope–tube interference, we selected a thin 10-Fr Salem sump OG tube. For stiffness, a guidewire was threaded into the tube via an 18-G side puncture ( Fig. 2 and Video 1 ), increasing column stiffness so the tube resists procedure-induced bending and tip migration. Olive oil was applied to the scope and tube to ease parallel movement.

**Fig. 1:**
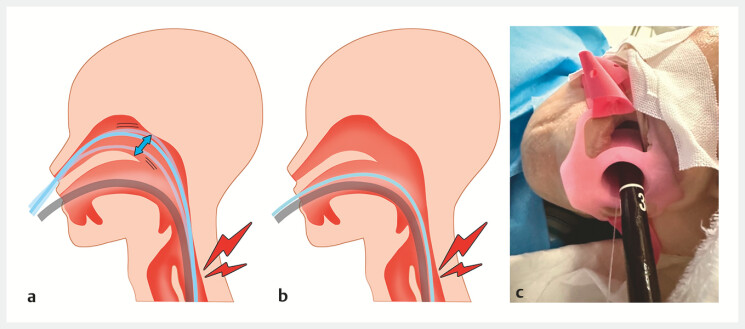
Nasal versus oral orogastric drainage routes for esophageal UESD. **a** Schematic of a nasogastric route with a long S-shaped course prone to bending, kinking, and tip displacement. **b** Schematic of the shorter, straighter oral route, reducing redundant the tube length and path curvature. **c** A clinical photograph showing an orogastric tube inserted outside the mouthpiece, alongside the endoscope, and secured at the right oral commissure. UESD, underwater endoscopic submucosal dissection [rerif].

**Fig. 2:**
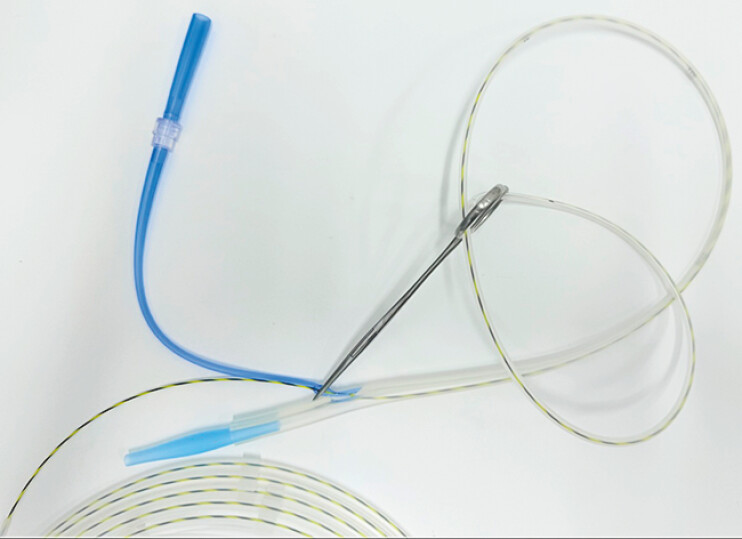
A guidewire-stiffened orogastric tube. An external view of a 10-Fr Salem sump OG tube with a guidewire inserted via an 18-G side puncture to increase stiffness.

Continuous gastric decompression via OG suction, together with the barrier function of the upper esophageal sphincter (UES), promotes a “one-way flow”: infused saline preferentially moves distally into the stomach and is recovered there by the OG tube rather than regurgitating orally ( Fig. 3 and Video 1 ). The intraoperative endoscopic view during UESD demonstrates a floating mucosal flap and clearly visible submucosal layer, illustrating the benefit of UESD in the esophagus ( Fig. 4 and Video 1 ). The tumor was completely resected within approximately 75 minutes.

**Fig. 3:**
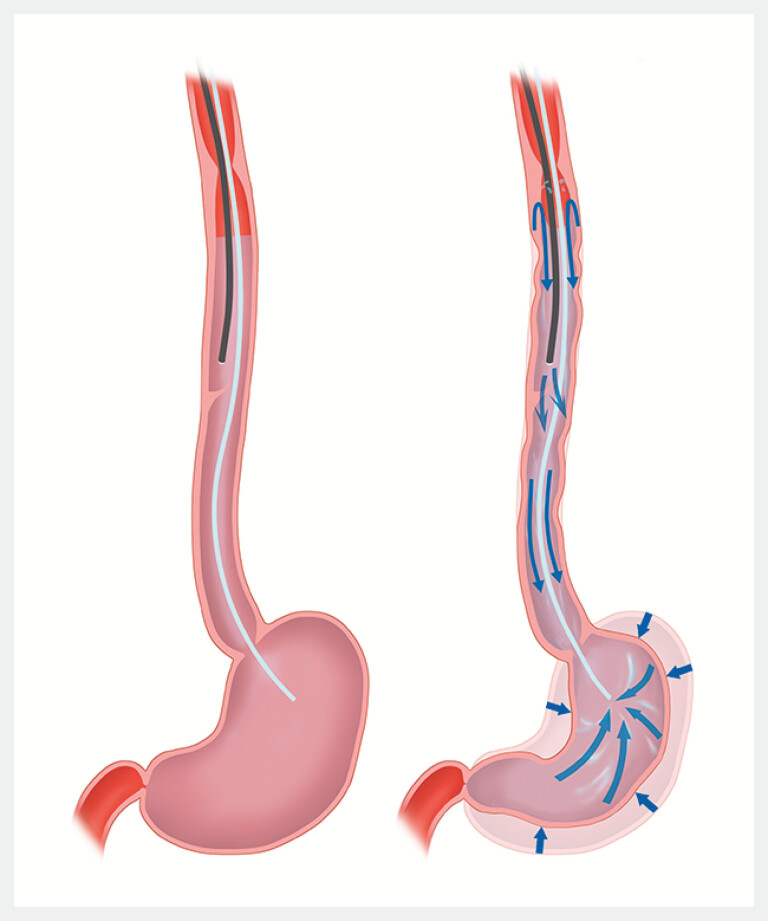
Schematic illustration of the “one-way flow” concept. Continuous gastric decompression via the orogastric tube and the barrier function of the upper esophageal sphincter directs infused saline distally into the stomach, where it is evacuated by the tube, thereby minimizing oral regurgitation and aspiration risk [rerif].

**Fig. 4:**
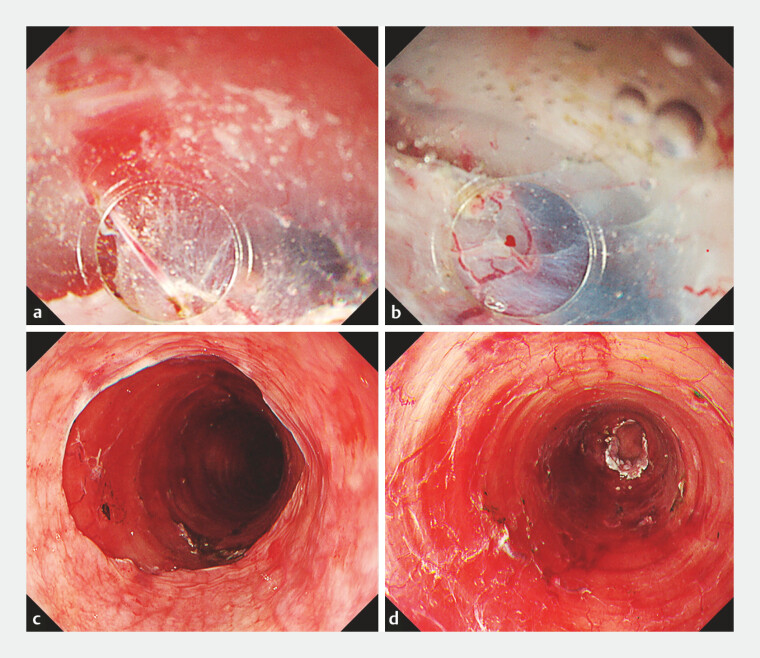
Representative intraoperative views of esophageal UESD. Sequential endoscopic images of a circumferential upper thoracic squamous cell carcinoma treated by UESD. **a, b** An intraoperative view during submucosal dissection demonstrating a floating mucosal flap and a clearly visualized submucosal layer, which facilitates precise dissection. **c, d** The final post-UESD ulcer bed. UESD, underwater endoscopic submucosal dissection.

The video details guidewire insertion into an orogastric tube, demonstrating the continuous drainage that constructs a “one-way flow.” This setup enables stable, non-intubated esophageal UESD and safe resection. UESD, underwater endoscopic submucosal dissection.Video 1

The total infused saline was 4,900 mL; 4,200 mL was evacuated via the OG tube and 500 mL via the endoscope. No gag reflex or oral regurgitation occurred, despite the proximal tumor edge being only 5 cm distal to the UES, and no adverse events, including aspiration pneumonia, were observed.

A previous report described fixing a nasogastric tip to the gastric wall with a clip and thread for continuous perfusion/retention 4 . In contrast, our non-fixed OG tube remained generally stable and could be adjusted whenever drainage seemed suboptimal. By combining gravity, gastric decompression, and the natural UES barrier to establish a one-way flow, this low-cost, reproducible setup may complement infusion-focused gel/underwater approaches and help extend UESD to the esophagus without intubation.

Endoscopy_UCTN_Code_TTT_1AO_2AG_3A

## References

[1] Yoshii S Akasaka T Hayashi Y “Underwater” endoscopic submucosal dissection: a novel method for resection in saline with a bipolar needle knife for colorectal epithelial neoplasia Surg Endosc 2018 32 5031 5036 10.1007/s00464-018-6278-x 30259162

[2] Nagata M Usefulness of underwater endoscopic submucosal dissection in saline solution with a monopolar knife for colorectal tumors (with videos) Gastrointest Endosc 2018 87 1345 1353 10.1016/j.gie.2017.11.032 29242059

[3] Sasaki S Nishikawa J Yamamoto K Effectiveness of underwater endoscopic submucosal dissection for a superficial cervical esophageal cancer Clin Endosc 2020 53 497 498 10.5946/ce.2020.031 32340085 PMC7403017

[4] Kowazaki Y Fukuda H Miwata T Nasogastric tube combined with thin therapeutic endoscope to facilitate esophageal endoscopic submucosal dissection Endosc Int Open 2024 12 E1196 E1198 10.1055/a-2421-9676 39411362 PMC11479792

